# Anterior distal tibial plafond-plasty for the treatment of posttraumatic ankle osteoarthritis with anterior translation of the talus

**DOI:** 10.1038/s41598-021-83946-y

**Published:** 2021-02-23

**Authors:** Xingchen Li, Yang Xu, Changjun Guo, Chonglin Yang, Yuan Zhu, Xiangyang Xu

**Affiliations:** Orthopaedic Department, Ruijin Hospital, Shanghai Jiaotong University School of Medicine, Shanghai, China

**Keywords:** Musculoskeletal system, Bone, Cartilage

## Abstract

Posttraumatic ankle osteoarthritis (OA) represents a significant challenge to orthopedic surgeons, especially in cases of anterior talar translation and concomitant impaction of the anterior distal tibial plafond. The aim of this study was to evaluate the clinical outcomes of an intra-articular osteotomy for the management of these patients. A total of 21 patients meeting our criteria were retrospectively reviewed. Sixteen patients sustained initial pilon fractures, while five patients had Weber type C ankle fractures. Anterior distal tibial plafond-plasty was performed to address the impaction and anterior translation of the talus. The American Orthopedic Foot and Ankle Society (AOFAS) hindfoot score and visual analog scale (VAS) score were utilized as clinical outcomes. The lateral talar station (LTS), tibial lateral surface (TLS) angle, tibial anterior surface angle and talocrural angle were evaluated pre- and postoperatively. The modified Kellgren-Lawrence score was used for the evaluation of sagittal ankle OA. The average age at surgery was 35 years, and the average follow-up duration was 34 months. The AOFAS hindfoot score increased from 26 to 71 (*p* < 0.01), and the VAS score improved from 7 to 2 (*p* < 0.01). The LTS improved from 9.0 to 2.3 mm (*p* < 0.01), and the TLS angle improved from 72° to 81° (*p* < 0.01). Of the 21 patients, 18 showed improvement in or no worsening of ankle OA on the sagittal plane, while 3 developed advanced ankle OA. A congruent ankle joint on the sagittal plane could be achieved by anterior distal tibial plafond-plasty. This is a valuable treatment option for the salvage of posttraumatic ankle OA with anterior translation of the talus.

## Introduction

Ankle fracture is the most common fracture around the foot and ankle. Although open reduction and internal fixation (ORIF) provides encouraging clinical outcomes in the treatment of displaced intra-articular ankle fractures, as shown in the literature^1,2^, posttraumatic ankle OA is still inevitable for over 20% of patients^3^. Tibial pilon fractures are high-energy injuries characterized by the presence of die-punch fragments of the articular surface^4^. The amount of energy and articular surface involvement are significantly correlated with posttraumatic ankle OA^5^. Failure to achieve anatomical reduction of the articular surface can lead to malunion of the fracture with articular step-off, which is associated with poor clinical results^6^.

The management of ankle fracture malunion can be challenging, especially in cases of malunited pilon fractures with high comminution of the distal tibial plafond. Any imperfect reduction of the articular fragments or metaphysis might cause step-off of the articular surface or angular deformity. A review of the literature showed that deformity of the distal tibial plafond on the coronal plane could be successfully treated with supramalleolar osteotomy^7–12^. The philosophy of supramalleolar osteotomy is to shift the load and redistribute the stress within the ankle joint by rearranging the alignment of the hindfoot.

However, most of these reports have focused on deformity correction on the coronal plane. There have been few studies about deformity correction on the sagittal plane. Sagittal plane deformity could change the ankle joint mechanics to a greater degree than coronal plane deformity^13,14^. According to a biomechanical study, a reduction in the contact area of the tibiotalar joint might result from anterior subluxation of the ankle joint, subsequently causing stress concentration within the ankle joint^15^. Scheidegger et al.^16^ reported a retrospective study of flexion osteotomy in a total of 39 patients with distal tibial recurvatum deformity. However, this technique was designed to correct extra-articular deformity on the sagittal plane.

In this study, we included a cohort of patients who developed ankle fracture malunion. The fractures were characterized by anterior translation of the talus with impaction of the anterior distal tibial plafond. A novel intra-articular osteotomy, called anterior distal tibial plafond-plasty, was performed to reconstruct the distal tibial plafond and realign the ankle on the sagittal plane. The purpose of this study was to investigate the corresponding clinical outcomes.

## Results

The average follow-up duration was 33.6 ± 8.3 (range, 19–46) months. The clinical outcomes and radiological parameters are listed in Table 1. Of the 21 patients, 18 showed improvement in or no worsening of ankle OA on the sagittal plane, while 3 developed advanced ankle OA. However, the patients were still satisfied with the clinical results and unwilling to undergo joint-sacrificing procedures. Three patients developed a superficial infection, which was successfully managed with antibiotics and regular dressing changes.

**Table 1:** Clinical and radiological outcomes.

	Preop (Mean ± SD)	Postop (Mean ± SD)	*p* Value
AOFAS	26.1 ± 19.8	71.3 ± 7.2	< 0.01*
VAS	7.3 ± 1.2	2.4 ± 1.1	< 0.01*
Plantarflexion, deg	21.2 ± 6.3	22.1 ± 8.2	0.62
Dorsiflexion, deg	7.9 ± 8.3	10.2 ± 5.8	0.15
LTS (mm)	9.0 ± 3.4	2.3 ± 1.7	< 0.01*
TLS (deg)	71.9 ± 4.2	81.2 ± 3.1	< 0.01*
TAS (deg)	86.0 ± 4.3	88.1 ± 1.8	0.03*
Talocrural angle (deg)	75.9 ± 4.0	76.4 ± 3.1	0.45
**Modified Kellgren–Lawrence score**			
I	0	2	0.32
II	7	9	
III	14	7	
IV	0	3	

To further analyze the factors associated with the clinical outcomes, multivariable linear regression was performed. We used the postoperative AOFAS hindfoot score as the dependent variable and included age, body mass index (BMI), latency time (time from initial to revision surgery), LTS, preoperative modified Kellgren-Lawrence score as 5 independent variables. The preoperative modified Kellgren-Lawrence score was the only predictor of clinical outcomes (*p* = 0.009 < 0.05; coefficient, -8.36). Patients with a lower preoperative grade of ankle OA tended to have better clinical outcomes. However, age (*p* = 0.61), BMI (*p* = 0.21), latency time (*p* = 0.81) and LTS (*p* = 0.36) were not associated with the clinical outcomes.

## Discussion

The clinical management of posttraumatic ankle OA remains a challenge. For patients with end-stage ankle OA, joint-sacrificing procedures seemed inevitable. Ankle fusion or total ankle arthroplasty could effectively relieve pain and improve function^17,18^. However, for selected patients with mild to mid-stage ankle OA, corrective osteotomy could be an alternative for salvage of the ankle joint. A review of the literature showed that supramalleolar osteotomy is recognized as an effective corrective procedure to address malalignment of the hindfoot on the coronal plane^7–12^.

However, few studies could be found about the management of deformity on the sagittal plane^4,19^. Patients included in this study were characterized as having anterior translation of the talus and impaction of the anterior distal tibial plafond. We felt that a series of intra-articular osteotomies of the distal tibial plafond (plafond-plasty) would help to achieve a congruent ankle joint on the sagittal plane. Bony correction of the distal tibial plafond realigns the ankle on the sagittal plane and provides anterior stability for the ankle joint, preventing anterior translation of the talus. Mann et al.^20^ reported a novel intra-articular osteotomy for the management of intra-articular varus ankle deformity. The varus ankle tilt deformity was improved significantly from 18° to 10° by this intra-articular medial opening wedge osteotomy of the distal tibia. Our surgical technique is analogous to that of both Rammelt and Zwipp^4^ and Guo et al.^21^, who presented novel intra-articular osteotomies (distal tibial plafond-plasty) to correct malunited pilon fractures with medial impaction on the coronal plane. A medial malleolar osteotomy was performed to help gain access to the articular step-off of the medial distal tibial plafond. A second osteotomy was performed above the impaction to reduce the step-off and achieve articular congruity. Although these were all intra-articular osteotomies on the coronal plane, their encouraging clinical outcomes inspired us to investigate whether an intra-articular osteotomy on the sagittal plane might play a role in addressing impaction on the sagittal plane in some patients. Rammelt et al.^4^ also presented an intra-articular osteotomy for pilon fractures on the sagittal plane; however, they gained access to the die-punch fragments between the anteromedial and anterolateral fragments and focused on articular reconstruction without the need to realign the ankle on the sagittal plane.

In our study, we had to address both anterior translation of the talus and distal tibial plafond impaction. Anterior distal tibial osteotomy was performed to expose the impaction, and then the articular surface step-off was reduced under direct vision. The anterior translation of the talus was addressed by extensive soft tissue release, and the anterior stability was reinforced by anatomical reduction of the distal tibial fragment with a buttress plate. According to our clinical data, the lateral talar station improved significantly from 9.0 to 2.3 mm. Three patients developed end-stage ankle OA; however, they were still satisfied with the operation and unwilling to undergo any further surgical intervention. Most of the patients benefited from our realignment procedure or at least did not develop worsening ankle OA. This could be the result of anatomical reduction of the ankle joint and redistribution of the pressure within the ankle joint. It was obvious that once malalignment was proven on X-rays, the earlier the revision surgery was performed, the less ankle joint cartilage would be destroyed and the more the patient would benefit from the realignment surgery^6^. Alignment should be considered as important as the articular cartilage itself. There might be some concerns about cartilage after correction; however, we believe that as long as the malalignment is addressed, there will be a favorable biomechanical environment for the articular cartilage. Especially in young and active patients with early- to mid-stage ankle OA, as long as the hindfoot malalignment has been addressed, they will gain the opportunity for ankle preservation or at least delay the need for a joint-sacrificing procedure.

**Figure 1:**
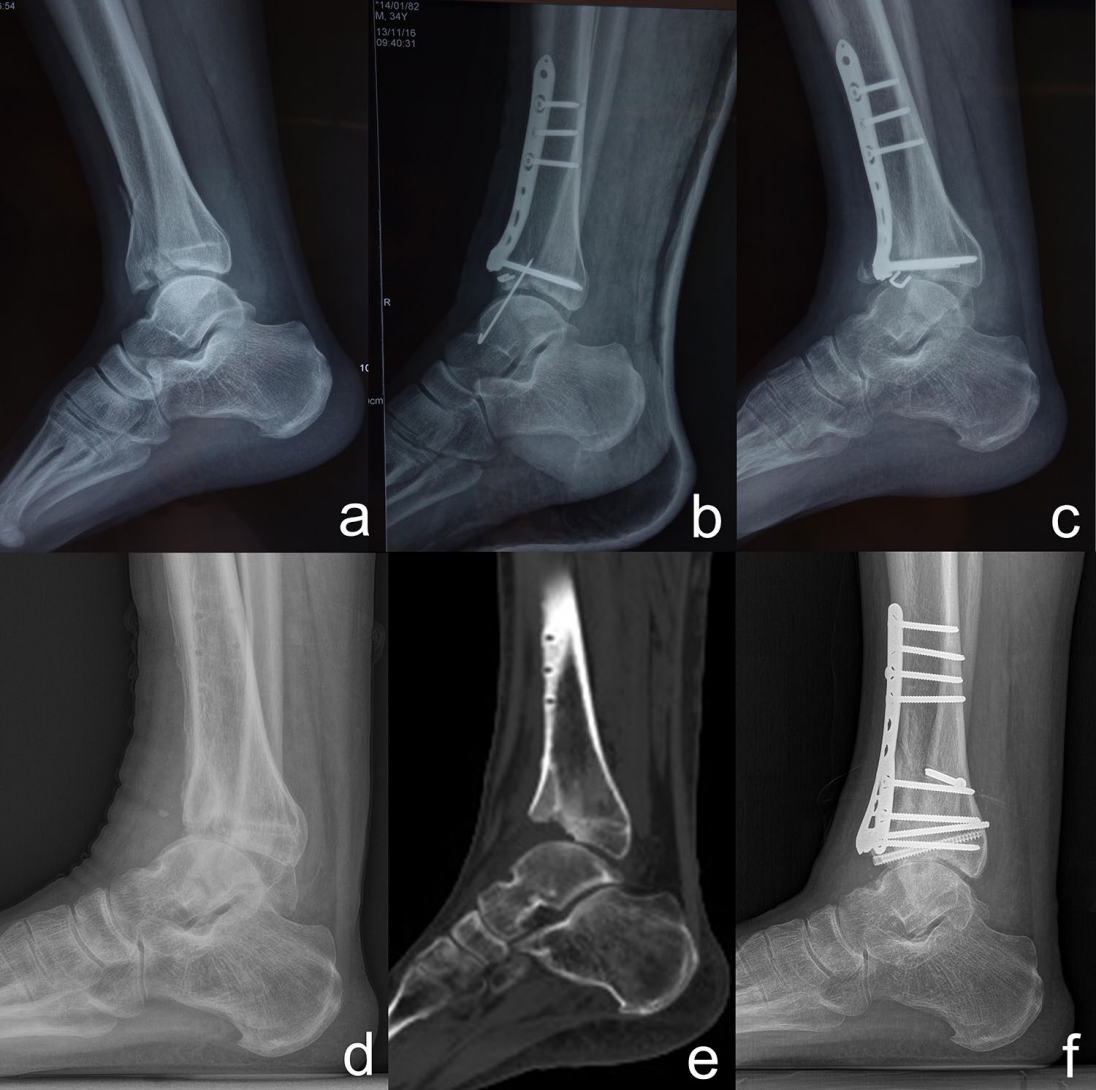
(**a**) A 36-year-old man sustained a pilon type B ankle fracture. (**b**) A congruent ankle joint and anatomical reduction of the fragments were observed on X-rays immediately after ORIF. (**c**) This patient gradually developed anterior subluxation of the ankle joint and impaction of the anterior distal tibial plafond. (**d–e**) Twelve months after the initial surgery, this patient visited our clinic for consultation because of worsening pain around the ankle. (**f**) At 16 months after the revision operation, alignment of the ankle was normal on the sagittal plane, and the patient was satisfied with the results of the operation.

In an attempt to understand the reason why the talus was anteriorly translated, we investigated the X-rays in emergency cases before and immediately after initial treatment. Impaction of the anterior distal tibial plafond or anterior dislocation of the ankle joint was seen in all in emergency cases. We assumed that the intact distal tibial plafond as well as the medial and lateral ankle ligaments provide stability for the ankle joint on the sagittal plane; pilon fractures cause impaction of the anterior distal tibial plafond, and failure to restore these anterior fragments intraoperatively, the loss of reduction postoperatively or nonunion might result in anterior distal tibial plafond impaction^4^, subsequently followed by anterior translation of the talus. Eleven of the sixteen pilon fractures in this cohort revealed anterior translation of the talus immediately after the initial operation, and the X-rays demonstrated inadequate reduction of the impacted fragments intraoperatively. Although a congruent ankle joint and adequate reduction were achieved in three patients, these patients still showed loss of the reduction of the anterior distal tibial plafond fragments after the operation and eventually developed anterior translation. This could be the result of nonunion of the anterior fragments (Fig. 1).

**Figure 2:**
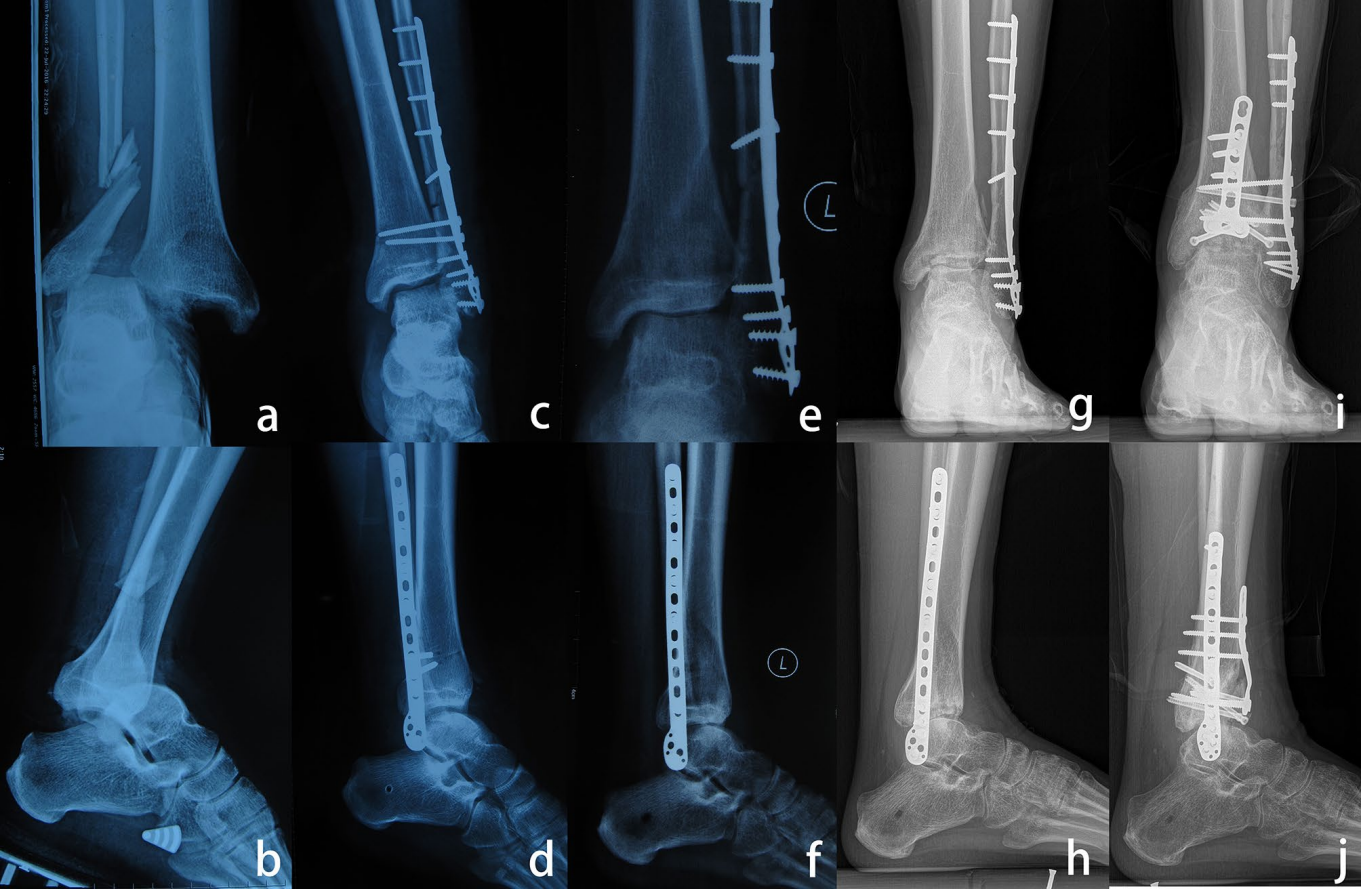
(**a,b**) A 34-year-old man sustained an open Weber type C ankle fracture. (**c,d**) ORIF was performed at 10 days after injury, and the distal tibia plafond was initially intact. (**e,f**) As the initial operation failed to reduce the fibula and distal syndesmosis anatomically, the talus was still anteriorly translated after the initial surgery. (**g,h)**. The anterior distal tibial plafond gradually became impacted as weightbearing was initiated. (**I,j**) Two years after realignment surgery, the ankle was normally aligned, and the patient was satisfied with the results of the operation.

Patients with Weber type C ankle fractures sustained complete rupture of the distal tibiofibular syndesmosis, and anterior subluxation of the ankle joint was common in emergency cases. All five patients with Weber type C ankle fractures in this cohort demonstrated subluxation of the ankle joint immediately after the initial operation, which could be the result of inadequate reduction of the syndesmosis, fibula, or posterior or medial malleolus. Interestingly, none of these patients initially showed distal tibial plafond fractures; however, they eventually developed impaction of the anterior distal tibial plafond. The pathology might involve cyclic loading and impaction of the talar dome onto the anterior distal tibial plafond (Fig. 2).

There were several limitations to this study. This was a retrospective study with a limited number of patients. Additionally, it analyzed short- to mid-term clinical results, and as ankle OA might develop over time, the study period might have been too early to determine this clinical outcome. The use of only the AOFAS hindfoot score for the evaluation of ankle function was also a limitation. Studies with a larger sample size, a longer follow-up period with postoperative CT scans, and more validated functional scores are needed to fully understand the clinical efficacy of this method.

## Materials and methods

### Inclusion and exclusion criteria

A retrospective cohort of consecutive patients with anterior translation of the talus and ankle OA between January 2015 and December 2018 was evaluated. This study was approved by the ethics committee of our hospital. The study was carried out in accordance with the Declaration of Helsinki. Informed consent was obtained from all individual participants of this study or their parents or guardians.

The patient inclusion criteria were as follows: (1) posttraumatic ankle OA with anterior translation of the talus and impaction of the anterior distal tibial plafond; and (2) grade 1 to 3 ankle OA according to the modified Kellgren-Lawrence score. The exclusion criteria were as follows: (1) grade 4 ankle OA; (2) acute or chronic infection; and (3) osteoporosis, age > 55 years old, diabetes, or peripheral vascular disease.

A total of 21 consecutive patients met our criteria. There were 12 males and 9 females, and their average age at the time of reconstructive surgery was 35.2 ± 9.4 (range, 16–48) years. The average BMI was 23.6 ± 2.9 (range, 18.7–31.2) kg/m^2^. The average latency time, i.e., the interval between the initial treatment and the revision surgery, was 7.8 ± 2.2 (range, 4–12) months. There were 4 open fractures and 17 closed fractures.

In a review of the X-rays in emergency cases before and immediately after the initial operation, 16 patients had pilon fractures (ten of them were AO/OTA type B pilon fractures, and the other six were AO/OTA type C pilon fractures), and the remaining 5 patients had Weber type C ankle fractures. While 11/16 patients with pilon fractures and 5/5 patients with Weber type C ankle fractures showed anterior translation of the talus immediately after the initial operation, 13/16 patients with pilon fractures and 0/5 patients with Weber type C ankle fractures showed anterior distal tibial plafond impaction immediately after the initial operation.

### Clinical and radiographic assessment

**Figure 3:**
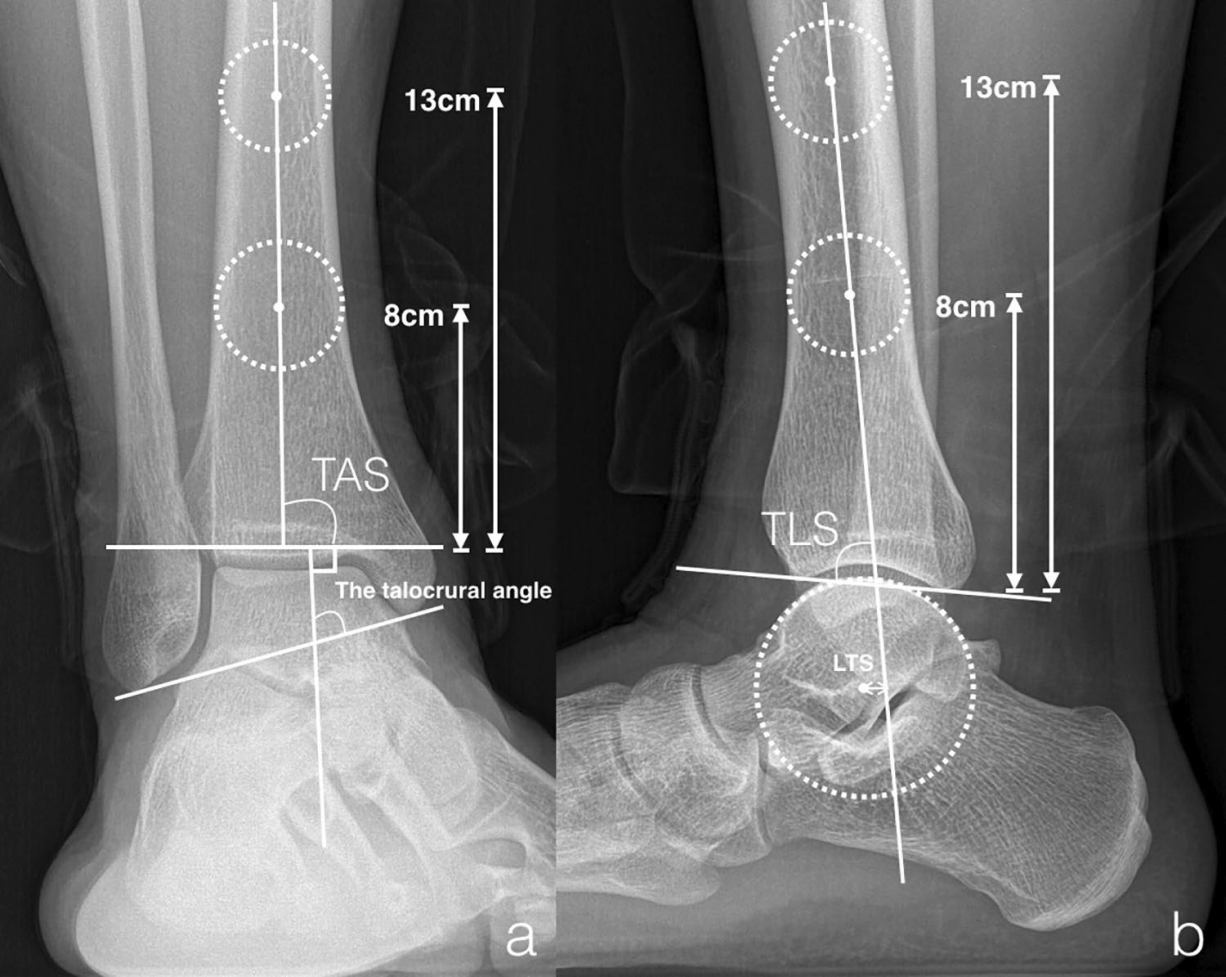
Radiographic measurement. (**a**) TAS, tibial anterior surface; (**b**) TLS, tibial lateral surface; LTS, lateral talar station.

A detailed review of both pre- and postoperative functional outcomes and radiological variables was performed. The functional outcome was evaluated using the AOFAS hindfoot score. Pain was measured by the VAS score. Weightbearing anteroposterior (AP) and lateral views of the affected ankle joint were obtained for evaluation pre- and postoperatively (Fig. 3). The lateral talar station (LTS) was defined as the distance of the center of rotation of a circle fitting the talar dome to the tibial axis line on the weightbearing lateral view of the ankle joint^14^. The tibial lateral surface (TLS) angle was formed by the angle between the mechanical axis of the tibia and a line connecting the most anterior and posterior margin of the tibial plafond. The tibial anterior surface (TAS) angle was formed by the angle between the mechanical axis of the tibia and the tibial plafond on the AP view^22^. The talocrural angle was constructed by drawing a perpendicular line downward from the tibial plafond surface, which was crossed by a line drawn connecting the tips of the medial and lateral malleoli^23^. The modified Kellgren-Lawrence score was utilized to evaluate ankle OA on the sagittal plane pre- and postoperatively^24^.

### Surgical technique

The aim of our realignment surgery was to preserve the ankle joint, while the principle of the surgery was to anatomically reduce the ankle joint, reconstruct the articular surface of the distal tibial plafond, and achieve a congruent and stable ankle joint. These aims could be achieved by corrective osteotomies, including the anterior distal tibial plafond-plasty, supramalleolar osteotomy, and fibula osteotomy, and soft tissue procedures, including medial and posterior release and medial and lateral ligament reconstruction.

The anterior approach was used for distal tibial plafond-plasty. The anterior distal tibial plafond and talar dome were fully exposed by opening the anterior joint capsule. A decision was made to preserve or fuse the ankle during the revision operation. If the cartilage on the distal tibial plafond was in fair condition, a joint-preserving procedure was performed.

For patients with prolonged anterior translation of the talus, adequate soft tissue release around the ankle is often mandatory to achieve anatomical reduction of the ankle joint^25^. Malreduction of the fibula and distal tibiofibular syndesmosis often occurs in patients with Weber type C ankle fractures. Extensive contracture or scar tissue was often anticipated at the syndesmosis and medial gutter. The adequate debridement and release of scar tissue at the syndesmosis was required to reduce the ankle joint.

**Figure 4:**
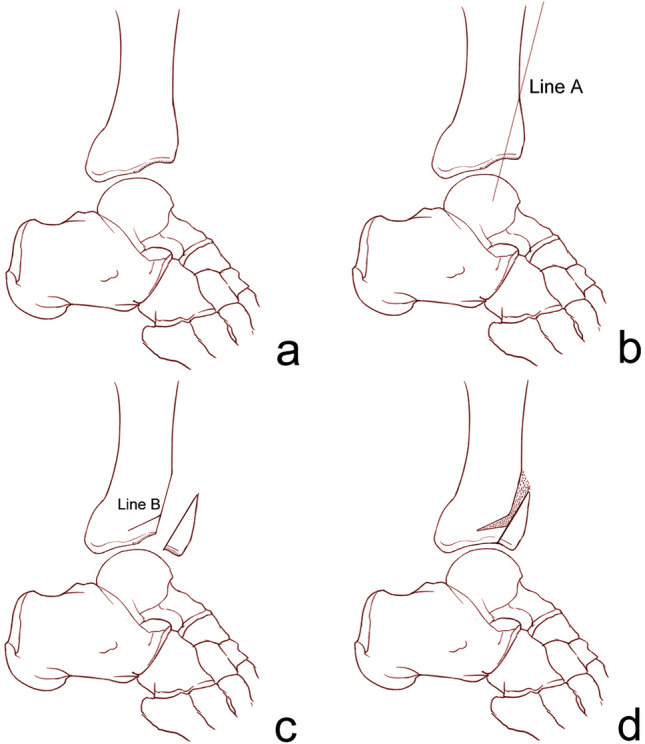
Schematic drawing of anterior distal tibial plafond-plasty. (**a**) Impaction of the anterior distal tibial plafond was noticed after the talus was reduced back to its normal position. (**b**) An anterior distal tibial osteotomy was performed along line A, from the superior anterior distal tibia to the apex of the impacted area. (**c**) A second osteotomy (line B) was performed just above the subchondral bone of the impacted articular surface. The articular surface step-off was reduced. (**d**) The distal tibial fragment was slid distally until a congruent ankle joint was achieved.

**Figure 5:**
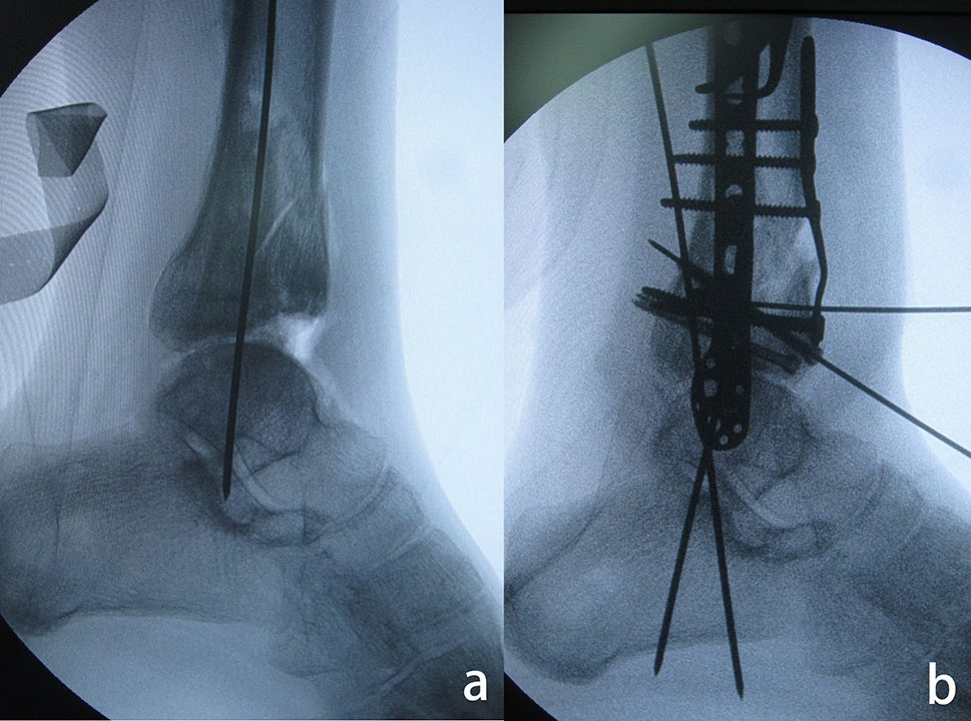
Anterior distal tibial plafond-plasty. (**a**) Impaction of the anterior distal tibial plafond was identified after anatomical reduction of the talus. (**b**) A congruent ankle joint was achieved. Screws and a buttress plate were utilized for fixation.

Immediately after the talus was reduced back to its normal position, a Kirschner wire was temporarily introduced to maintain the reduction. Another Kirschner wire was used to mark the direction of the osteotomy from superior anterior to the apex of the impacted area. Intraoperative fluoroscopy could facilitate confirmation of the Kirschner wire direction. The anterior distal tibial plafond-plasty was performed with an oscillating saw parallel to the Kirschner wire. A sagittal cut might be needed to separate the fragment from the medial malleolus, and it could be made just at the medial corner. Then, a wide osteotome was used to open the osteotomy to fully expose the impacted articular surface. A second osteotomy was required just above the impacted subchondral bone, and a small chisel was utilized to meticulously reduce the step-off of the articular surface under direct vision. An allograft was used to fill the osteotomy gap. The anterior distal tibial fragment was slid distally, and the osteotomy was opened until a congruent ankle joint was achieved, as confirmed intraoperatively under fluoroscopy. The allograft was reinserted into the osteotomy gap, and Kirschner wires were utilized for temporary fixation of the anterior distal tibial fragment. Excessive bone was resected to match the contour of the anterior tibial surface. Cannulated screws and buttress plates were utilized to secure the osteotomy (Figs. 4, 5).

**Figure 6:**
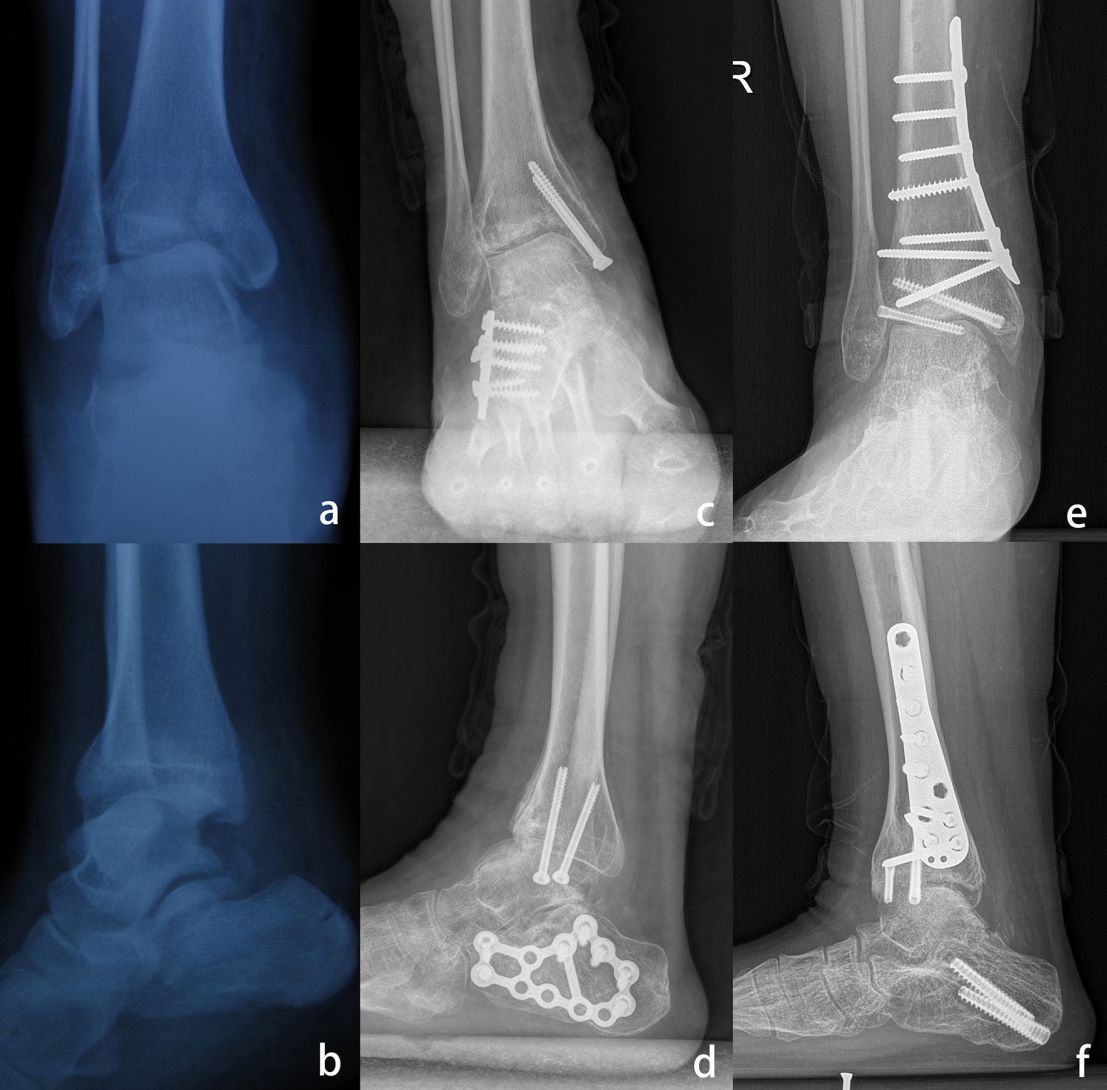
(**a,b**) A 16-year-old girl had a pilon type B ankle fracture and calcaneal fracture. (**c,d**) Open reduction and internal fixation was performed; however, she developed malunion of the distal tibial plafond fracture. The TAS angle was 74°, and the distal tibial plafond was impacted by anterior translation of the talus. (**e,f**) Corrections on both the sagittal and coronal planes of the distal tibial plafond were performed, with excellent clinical outcomes three years after revision surgery.

Malunion of the fibula is also a common scenario, often involving shortening or rotation of the lateral malleolus^26,27^. In this study, a fibular osteotomy was performed in 10 patients. A horizontal fibular osteotomy was used because it was the easiest way to correct both shortening and rotational deformity of the fibula^6^. An allograft was inserted if the fibula needed lengthening. In addition, a supramalleolar osteotomy was performed in 6 patients; it was indicated to correct the TAS angle to 85° to 90° (Fig. 6)^28^. Ligament reconstruction would provide extra stability for the ankle joint. We routinely assessed ankle stability intraoperatively after bony corrections. Lateral ligament reconstruction was performed in 12 patients. The modified BrostrÖm procedure with suture anchors was used in 4 patients, while lateral ligament reconstruction was performed with allografts in the remaining 8 patients with inadequate soft tissue. Three patients needed extensive soft tissue release extending to the medial ankle ligaments, and medial ligament reconstruction with suture anchors was mandatory to enhance the stability of the ankle joint following plafond-plasty.

### Postoperative rehabilitation

The ankle was stabilized in the neutral position with a well-padded, short leg splint. The wound was inspected on the second day after the operation, and a walking boot was applied. Nonweightbearing range of motion exercise was initiated at one or two weeks after the operation when tolerated. Partial weightbearing with the protection of the walking boot was encouraged at six weeks after the operation with gradual progression to full weightbearing at three months after the operation.

### Statistical analysis

Statistical analyses were performed using IBM SPSS Statistics, version 23.0 (IBM Corp., Armonk, New York). The normality of the data, including the pre- and postoperative radiological parameters and clinical outcomes, was tested using the K-S test. For data showing normality, Student’s t test was performed. For data not showing normality, Wilcoxon’s signed rank test was used. Multivariable linear regression was utilized to determine the risk factors for clinical results. Significance was set at *p* < 0.05.

## Ethics approval

This study was approved by the ethics committee of Ruijin Hospital, Shanghai JiaoTong University School of Medicine.

## Consent to participate

Informed consent was obtained from all individual participants included in the study. For participants aged less than 18 years, a statement of informed consent was obtained from the parents or guardians of the participants.

## Consent for publication

Written consent to publish the related images or clinical details was provided by participants included in the study.

## Data Availability

All data supporting our findings are contained within the manuscript. All data in this study are freely available to any researcher for noncommercial purposes.
